# Thyroid hormone synthesis continues despite biallelic thyroglobulin mutation with cell death

**DOI:** 10.1172/jci.insight.148496

**Published:** 2021-06-08

**Authors:** Xiaohan Zhang, Aaron P. Kellogg, Cintia E. Citterio, Hao Zhang, Dennis Larkin, Yoshiaki Morishita, Héctor M. Targovnik, Viviana A. Balbi, Peter Arvan

**Affiliations:** 1 Division of Metabolism, Endocrinology and Diabetes, University of Michigan, Ann Arbor, Michigan, USA.; 2 Universidad de Buenos Aires, Facultad de Farmacia y Bioquímica, Departamento de Microbiología, Inmunología, Biotecnología y Genética/Cátedra de Genética, Buenos Aires, Argentina.; 3 Consejo Nacional de Investigaciones Científicas y Técnicas, Universidad de Buenos Aires, Instituto de Inmunología, Genética y Metabolismo, Buenos Aires, Argentina.; 4 Division of Diabetes, Department of Internal Medicine, Aichi Medical University, Nagakute, Japan.; 5 Department of Endocrinology and Growth, Hospital de Niños Sor María Ludovica, La Plata, Argentina.

**Keywords:** Endocrinology, Thyroid disease

## Abstract

Complete absence of thyroid hormone is incompatible with life in vertebrates. Thyroxine is synthesized within thyroid follicles upon iodination of thyroglobulin conveyed from the endoplasmic reticulum (ER), via the Golgi complex, to the extracellular follicular lumen. In congenital hypothyroidism from biallelic thyroglobulin mutation, thyroglobulin is misfolded and cannot advance from the ER, eliminating its secretion and triggering ER stress. Nevertheless, untreated patients somehow continue to synthesize sufficient thyroxine to yield measurable serum levels that sustain life. Here, we demonstrate that *TG^W2346R/W2346R^* humans, *TG^cog/cog^* mice, and *TG^rdw/rdw^* rats exhibited no detectable ER export of thyroglobulin, accompanied by severe thyroidal ER stress and thyroid cell death. Nevertheless, thyroxine was synthesized, and brief treatment of *TG^rdw/rdw^* rats with antithyroid drug was lethal to the animals. When untreated, remarkably, thyroxine was synthesized on the mutant thyroglobulin protein, delivered via dead thyrocytes that decompose within the follicle lumen, where they were iodinated and cannibalized by surrounding live thyrocytes. As the animals continued to grow goiters, circulating thyroxine increased. However, when *TG^rdw/rdw^* rats age, they cannot sustain goiter growth that provided the dying cells needed for ongoing thyroxine synthesis, resulting in profound hypothyroidism. These results establish a disease mechanism wherein dead thyrocytes support organismal survival.

## Introduction

In the body, the circulating thyroid hormone, thyroxine (also known as T_4_) originates exclusively from biosynthesis within the thyroid gland. T_4_ biosynthesis occurs by a common mechanism in all vertebrates. Specifically, a monolayer of thyrocytes (also known as thyroid follicular epithelial cells) surrounds a central apical (extracellular) lumen, into which thyrocytes deliver a nearly pure secretion of thyroglobulin (Tg, encoded by the *TG* gene; ref. 1), which comprises ≥50% of the total protein of the thyroid gland (2). Thyrocytes exhibit a polarized distribution of plasma membrane enzymes/activities that coordinate thyroid peroxidase–catalyzed apical iodination of extracellular protein in the luminal cavity (3). Iodination of various tyrosine residues on secreted Tg (4) triggers the formation of T_4_ intramolecularly within the Tg protein (5, 6) prior to endocytic reentry of the hormone-containing protein into surrounding thyrocytes for lysosomal digestion, resulting in the proteolytic liberation and release of T_4_ from the basolateral membrane of thyrocytes to the bloodstream (7).

The first 3-dimensional atomic structure of human Tg has recently been reported (8). Already 227 different *TG* gene mutations have been found to be linked to congenital hypothyroidism (9); as far as is known, essentially all of the structurally defective Tg mutants are entrapped in the endoplasmic reticulum (ER), causing thyrocyte ER swelling and ER stress (10). Susceptibility to the many different pathogenic mutations is in part explained by the large and complex structure of the Tg protein (8), including its multiple repeat domains bearing internal disulfide bonds, and concluding with the cholinesterase-like (ChEL) domain (10). The C-terminal ChEL domain of Tg has no direct effect on thyroidal iodination machinery, but it (a) shares a similar structure with other ChEL family members (11), (b) provides information necessary and sufficient for the noncovalent homodimerization needed for intracellular transport (12), (c) functions as an intramolecular chaperone required to stabilize the folded structure of upstream repeat domains of Tg (13), and (d) provides its own hormonogenic iodination site (14). A number of patients have been reported with homozygous mutation in the Tg-ChEL domain (e.g., Tg-W2346R or Tg-G2322S; in the UNIPROT P01266 numbering system this would need to include the 19-residue signal peptide) with congenital hypothyroidism (15, 16).

In years past, cases of congenital hypothyroidism could go undiagnosed in early life due to insufficient neonatal screening (17). Classic studies of Marine and Lenhart showed that thyroid hyperplasia is induced as a consequence of primary hypothyroidism (experimentally induced following partial thyroidectomy or iodide deficiency) in animals (18) or humans (19); i.e., the endocrine feedback of primary hypothyroidism results in chronically upregulated pituitary secretion of thyroid-stimulating hormone (TSH), and such chronic stimulation contributes to exuberant growth of the thyroid gland (20–22). Therefore, patients with biallelic *TG* mutations would be expected to present, ultimately, with goiter. Interestingly, however, by linkage analysis, variants of the *TG* gene are linked to human hypothyroidism with or without thyroid goiter (23). Why hypothyroid patients with biallelic *TG* mutation (and no defect in TSH response) would not develop a goiter is unknown, although in the clinical setting, the understanding of goiter development is often confounded in patients who may or may not have received exogenous T_4_ treatment (24–26).

On the one hand, increased goiter growth might help to overcome genetic or acquired inefficiency of thyroid hormone synthesis (27); on the other, growth of a large goiter in iodine deficiency has been proposed to be a maladaptation (28). In either case, there has been a fundamental knowledge gap in understanding how untreated patients bearing pathogenic, biallelic *TG* mutations could possibly be capable of synthesizing endogenous T_4_.

Chronic, unremitting and unresolved ER stress is a factor that can promote cell death (29–34). Chronic ER stress is known to occur in the thyrocytes of *TG^cog/cog^* (congenital goiter) mice (encoding Tg-L2263P), which are famous for their hyperplastic goiter — and also in *TG^rdw/rdw^* (rat dwarf) rats (35) that do not develop a goiter (36). In all cases of biallelic *TG* mutation, it is thought that massive quantities of mutant Tg protein are blocked in forward advance from the ER, as in the *TG^cog/cog^* and *TG^rdw/rdw^* thyroid glands, triggering a dramatic ER stress response that is also seen in the thyroid glands of patients with this disease (35, 37–39). Thyrocyte cell death has never been considered in goitrous *TG^cog/cog^* mice or humans with biallelic *TG* mutations, but in *TG^rdw/rdw^* rats we posited that thyroid follicular cell death might block the development of goiter (40).

Importantly, untreated *TG^cog/cog^* mice spontaneously increase their levels of serum T_4_, paralleling growth of the thyroid gland, ultimately achieving nearly normal levels (41). With this in mind, in this report we have analyzed both rodent thyroid glands and those of individuals expressing biallelic *TG* missense mutations that render Tg incapable of forward trafficking from the ER. Remarkably, we found that thyrocyte cell death and disintegration within the thyroid follicle lumen provides the Tg substrate needed for synthesis of endogenous T_4_. The life of untreated individuals depends on this unusual mechanism of endogenous T_4_ synthesis, as even a brief exposure of such animals to antithyroid drugs is lethal. Most remarkably, we have uncovered compelling evidence that, in this disease, goiter growth is needed to provide an ongoing supply of dead cells so that thyroid hormonogenesis can be sustained.

## Results

### Mutant mice and humans bearing biallelic TG missense mutations endogenously synthesize T_4_ using substrate derived from dead thyrocytes.

**Figure 1:**
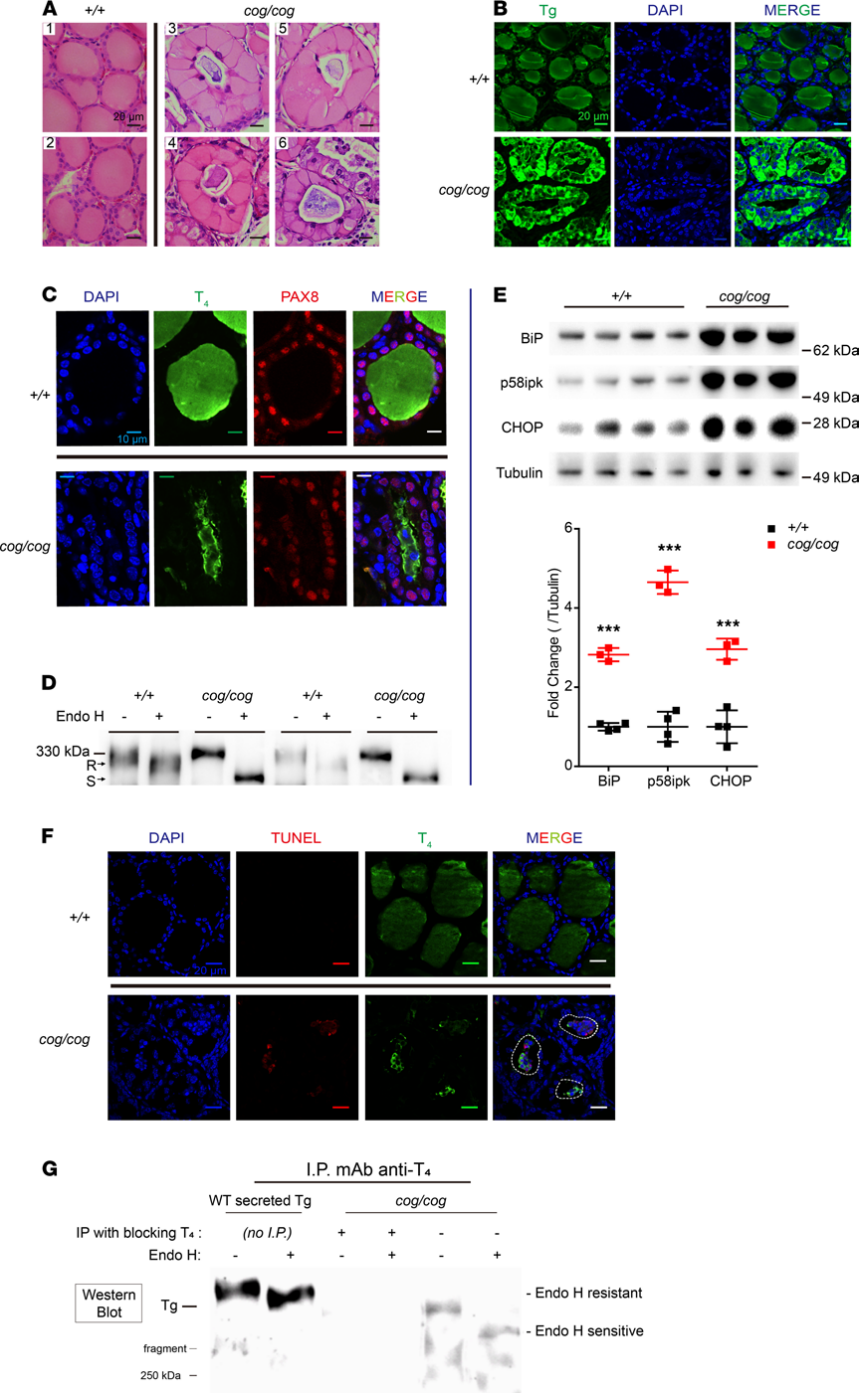
ER stress, cell death, and T4 synthesis in *TG^cog/cog^* mice. (**A**) Representative H&E-stained images of WT and *TG^cog/cog^* thyroids (*n =* 6 animals/group), showing thyrocyte distention with apically displaced nuclei in *TG^cog/cog^* mice compared with a thin monolayer of thyrocytes in WT (^+/+^) mice. Scale bars: 20 μm. (**B**) Representative anti-Tg immunofluorescence in thyroid glands of WT and *TG^cog/cog^* mice (*n =* 8 animals/group), with DAPI counterstain. Scale bars: 20 μm. (**C**) Representative immunofluorescence of T4-containing protein in thyroid follicles of WT or *TG^cog/cog^* mice (*n =* 6 animals/group). Thyrocytes are highlighted by PAX8-positive nuclear transcription factor with DAPI counterstain. Scale bars: 10 μm. (**D**) Endoglycosidase H digest of thyroid homogenates before SDS-PAGE and Tg Western blotting from WT and *TG^cog/cog^* mice (*n =* 3 animals/group; 2 of each kind shown). R, endoglycosidase H resistant; S, endoglycosidase H sensitive. (**E**) Top: Western blotting of BiP, p58ipk, and CHOP in the thyroids of *TG^cog/cog^* mice (*n =* 3–4; each lane represents 1 animal). Bottom: Quantitation of bands (normalized to tubulin), shown as mean ± SD. ***P < 0.001 (unpaired 2-tailed Student’s *t* test). (**F**) Representative TUNEL staining and immunofluorescence of T4-containing protein with DAPI counterstain in thyroid sections of WT and *TG^cog/cog^* mice (*n =* 7 animals/group). For clarity, in the merged image from *TG^cog/cog^* mice, a dashed white line delimits the thyroid follicle lumen. Scale bars: 20 μm. (**G**) Thyroid homogenate from *TG^cog/cog^* mice (*n =* 3) was immunoprecipitated with mAb anti-T4 in the presence or absence of T4 competitor, followed by either mock digest or Endo H digest and SDS-PAGE plus immunoblotting with mAb antibody that recognizes intact Tg. As a positive control, WT Tg secreted from PCCL3 (rat) thyrocytes was digested for Endo H resistance. The T4-containing Tg protein of *TG^cog/cog^* mice was entirely Endo H sensitive. The position of the 250 kDa molecular weight marker is shown.

With age, hypothyroid *TG^cog/cog^* mice (expressing homozygous Tg-L2263P) spontaneously increase their serum T_4_ to eventually reach nearly normal levels (41), which is perplexing because the mutant *cog*Tg protein cannot exit the ER (37, 38). We performed routine histology of WT and *TG^cog/cog^* mouse thyroid glands. Whereas thyroid follicles from WT mice showed the normal epithelial monolayer of thyrocytes surrounding an abundant proteinaceous extracellular lumen (Figure 1A, left), thyrocytes from *TG^cog/cog^* mice exhibited enormous intracellular distention (comprising massive ER expansion, ref. 42), with nuclei abnormally “pushed” into the apical cytoplasm (Figure 1A, right). Immunofluorescence of Tg in the thyroid follicles of WT mice revealed densely packed Tg protein within the extracellular lumen (Figure 1B, top), whereas in *TG^cog/cog^* mice, Tg was detectable in an abnormal, patchy distribution in the follicle lumen (Figure 1B, bottom). On the one hand, one might expect to find Tg in the follicle lumen, because *TG^cog/cog^* mice do produce T_4_, and Tg is the protein from which T_4_ is synthesized (10). Indeed, detectable T_4_-containing protein was generated (in the lumen of 92.4% ± 10.9% of *TG^cog/cog^* thyroid follicles [SD, *n =* 7 animals]), surrounded by a ring of thyrocytes (nucleated cells positive for the thyrocyte-specific transcription factor, Pax8; Figure 1C, bottom). Thus, in *TG^cog/cog^* mice, mutant Tg can reach the lumen of thyroid follicles, in which T_4_ is synthesized. On the other hand, mutant Tg is not thought to be competent for anterograde transport from the ER (37). For each Tg molecule that successfully undergoes anterograde transport from the ER, approximately two-thirds of the N-glycans on each molecule acquire Golgi sugar modifications (43, 44), enabling those glycans to acquire resistance to digestion with endoglycosidase H (Endo H; ref. 45). We confirmed that in WT mice, nearly all thyroidal Tg molecules (which, in the steady-state, reside primarily in the extracellular follicle lumen) have acquired Golgi-based “complex” N-glycans and thus have become Endo H resistant (Figure 1D). In contrast, in *TG^cog/cog^* thyroid glands, no Tg acquired Endo H resistance (and instead Tg remained completely Endo H sensitive, Figure 1D). These data indicate that no *cog*Tg undergoes intracellular trafficking to the Golgi complex. Although exosomes cannot convey Endo H–sensitive protein directly from the ER to the extracellular space (46, 47), the foregoing data do suggest that in the *TG^cog/cog^* thyroid gland, Tg must arrive in the lumen of thyroid follicles via a delivery mechanism other than the conventional secretory pathway. Interestingly, the patchy distribution of mutant Tg in the follicle lumen appeared to be associated with additional cellular material, including nuclear chromatin (Figure 1, A and B).

The synthesis of misfolded Tg is accompanied by ER stress (38, 48–51). Ongoing ER stress in the thyroids of *TG^cog/cog^* mice was demonstrable (Figure 1E), with a dramatic elevation of ER stress markers, including the ER hsp70 chaperone BiP (52) and its cochaperone p58ipk (encoded by DNAJC3; ref. 53). Additionally, ER stress–induced upregulation of CHOP (Figure 1E) can promote cell death (54, 55). These data (and additional evidence shown below) led us to consider that T_4_ synthesis in *TG^cog/cog^* mice might be based on mutant Tg being delivered to the thyroid follicle lumen via thyrocyte cell death. Such a possibility is not without precedent; indeed, upon cell death in other epithelia, including renal tubular epithelial cells, mammary epithelial cells, and bronchial epithelial cells, dead cells are extruded selectively to the apical side of the epithelium (56–58), which, in the thyroid, would correspond to the follicular lumen.

In the thyroids of *TG^cog/cog^* mice, we could detect abnormal nuclear material in the lumen of 74.0% ± 22.8% (SD, *n =* 4 animals) of thyroid follicles, including DAPI staining, suggesting karyolysis and karyorrhexis — swollen nuclei retained in dead-cell ghosts with less intense DAPI staining, consistent with a gradual disintegration of dead cells and their chromatin. Images of cell ghosts revealed positivity for CHOP (detected in 33% ± 9.7% of thyroid follicles, Supplemental Figure 1A); cleaved caspase-3 (an executioner caspase) was detected in 30% ± 11.4% (SD, *n =* 4 animals) of total follicles (Supplemental Figure 1B) and was positive by TUNEL staining, indicating cleaved DNA (Figure 1F, a dashed line drawn on the merged image highlights the apical luminal cavity). Crucially, more than 96% of TUNEL-positive cell ghosts were associated with the presence of T_4_ synthesis (*n =* 4 animals; see Figure 1F). No DAPI-positive, CHOP-positive, cleaved caspase-3–positive, or TUNEL-positive cells were observed within the thyroid follicle lumen of WT mice (Figure 1F and Supplemental Figure 1, A and B).

Some of the T_4_ synthesized in Tg can occur within small peptide regions that do not require the native globular structure of the entire molecule (8, 59). In pilot studies using a recently developed assay for thyroid hormone formation after in vitro iodination (14) of transfected cell lysates, we observed that ER-entrapped recombinant mutant *cog*Tg and *rdw*Tg (described below) have the potential to serve as substrate for T_4_ synthesis. Immunoblotting of unpurified thyroid homogenates with anti-T_4_ to identify T_4_-containing proteins revealed the major Tg hormone–containing fragment (~250 kDa, ref. 38) and its degradation products (7) in WT mouse thyroid tissue, whereas *TG^cog/cog^* thyroid glands did not immediately reveal a clear predominant species (Supplemental Figure 1C, lanes 2–4). We selectively concentrated T_4_-containing protein from *TG^cog/cog^* thyroid tissue by immunoprecipitation with anti-T_4_, followed by immunoblotting of the recovered samples with a mAb that specifically favors recognition of intact Tg (epitope located between Tg residues 1000 and 1100). As expected, when no tissue sample was included in the anti-T_4_ immunoprecipitation, no T_4_-containing Tg protein was recovered (Supplemental Figure 1C, lane 8). However, both WT (Supplemental Figure 1C, lane 7) and *TG^cog/cog^* thyroid glands clearly demonstrated Tg bearing T_4_ (Supplemental Figure 1C, lanes 9–11). Moreover, whereas secreted WT Tg was entirely Endo H resistant, the Tg that was specifically immunoprecipitated from *TG^cog/cog^* thyroid tissue by virtue of its T_4_ content was still fully Endo H sensitive (Figure 1G). Thus, albeit inefficient, in the thyroid glands of untreated *TG^cog/cog^* mice, T_4_ is synthesized in vivo on mutant Tg protein that has never traversed the Golgi complex.

**Figure 2:**
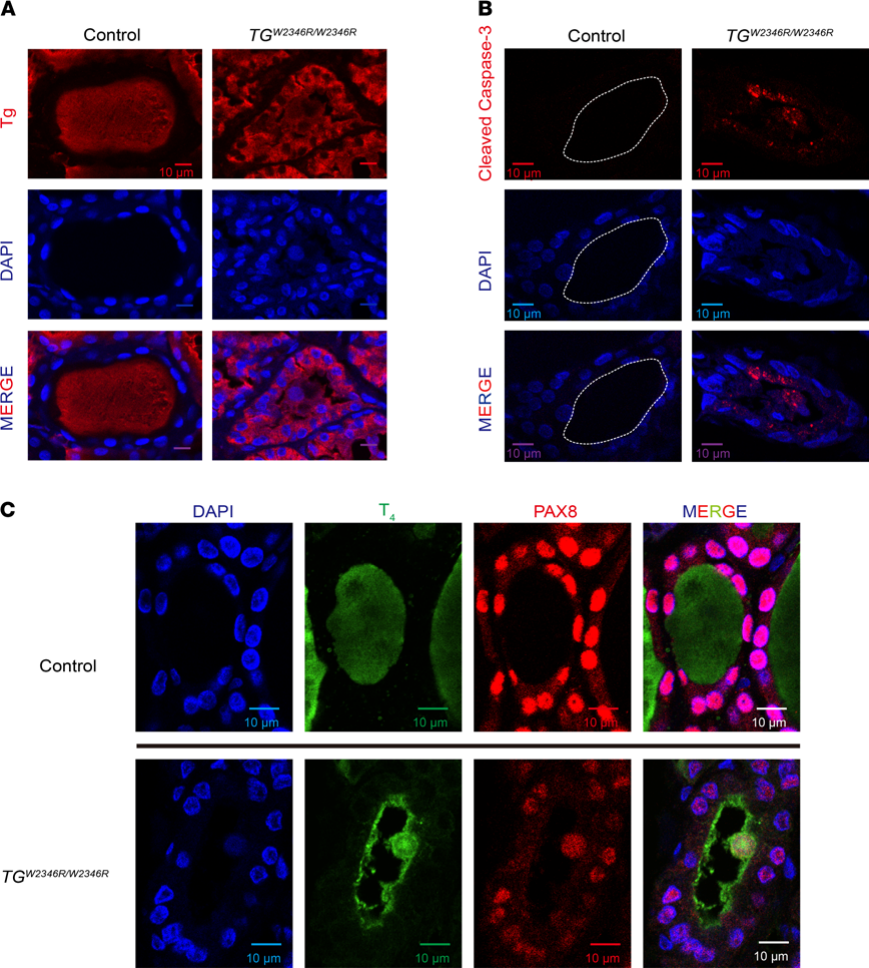
Tg and T4 synthesis in a homozygous patient bearing *TG^W2346R/W2346R^*. (**A**) Anti-Tg immunofluorescence (red) with DAPI counterstaining (blue) of human thyroid sections from a patient bearing *TG^W2346R/W2346R^* and a representative unaffected (Control) individual (*n =* 3). The diseased thyroid gland shows abnormal accumulation of intracellular Tg but also shows Tg in a patchy distribution in the thyroid follicle lumen. Scale bars: 10 μm. (**B**) Anti-cleaved caspase-3 immunofluorescence (red) with DAPI counterstaining (blue) in the thyroid gland of the individuals from **A**. For clarity, a dashed white line delimits the thyroid follicle lumen in the control (in which cleaved caspase-3 is not seen). Scale bars: 10 μm. (**C**) Immunostaining of T4-containing protein (green) in thyroid follicles from the individuals in **A**. Thyrocyte identity is confirmed by PAX8-positive nuclei (red) with DAPI counterstain (blue). Scale bars: 10 μm.

Similar to *TG^cog/cog^* mice, a patient bearing homozygous Tg-W2346R in the ChEL domain developed a large hypothyroid goiter, leading to thyroidectomy (15). Histological analysis of the patient’s thyroid tissue indicated the presence of abnormal nuclear and cytoplasmic profiles in follicle lumen (Supplemental Figure 2A). Cross sections of the patient’s goiter were immunostained with anti-Tg. Unlike the normal human thyroid (Figure 2A), more than 50% of thyroid follicle lumina contained the mutant Tg in an abnormal, patchy distribution that was associated with cellular material, including nuclei (Figure 2A), similar to that seen in *TG^cog/cog^* mice. Additionally, 49% of the patient’s thyroid follicles exhibited detectable cleaved caspase-3 (Figure 2B, an inner dashed line highlights the apical luminal cavity in the WT control, which is negative for cleaved caspase-3) and abnormal, weakly DAPI-positive material in the thyroid follicle lumen, consistent with various stages of nuclear disintegration (Figure 2). Indeed, residual positive signal for the Pax8 transcription factor was detectable (Figure 2C) — albeit usually at a lower level than that seen in surrounding living follicular cells (but clear enough to identify the dead cells as thyrocytes). Remarkably, ongoing T_4_ synthesis was detected in all of the follicles containing dead thyrocytes, with evidence suggesting endocytic recapture of T_4_-containing protein in an apically concentrated ring near the boundary between living thyrocytes and the luminal cavity (Figure 2C). Indeed, T_4_-containing substrate was prominently seen on cytoplasmic protein adjacent to the nuclei of TUNEL-positive dead thyrocytes. Additionally, the dead-cell ghosts were cannibalized (endocytosed) by surrounding live-cell neighbors, highlighting a positive ring of T_4_- immunostaining lining the apical region of live thyrocytes (Supplemental Figure 2, B–D). Together the data, both in rodents and humans with mutant Tg, indicate a pathological salvage mechanism of T_4_ synthesis that is built not upon Tg secretion but upon mutant Tg reaching the follicle lumen via exfoliation of ER-stressed, dead thyrocytes.

### In hypothyroidism with biallelic mutant TG, thyroid cell mass is the critical factor regulating thyroid hormone synthesis.

**Figure 3:**
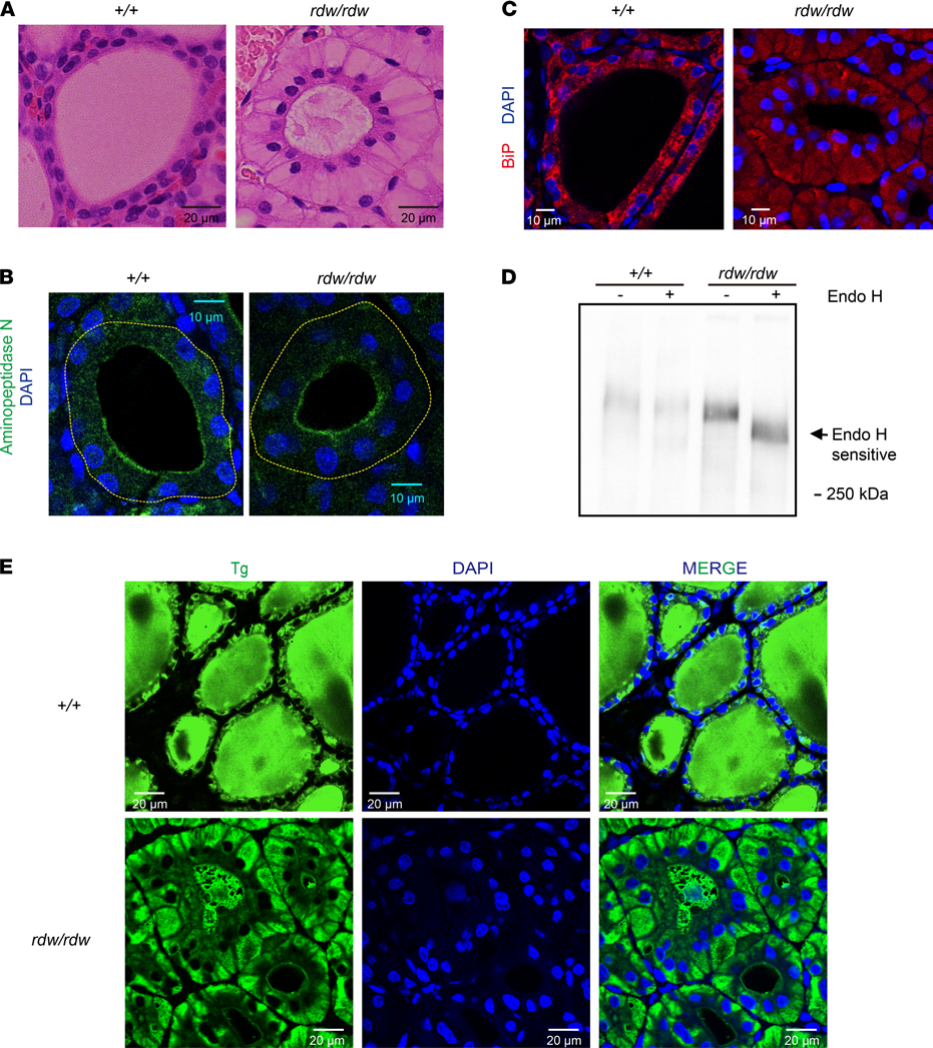
Tg is entrapped in the ER, yet it reaches the thyroid follicle lumen in *TG^rdw/rdw^* rats. (**A**) Representative H&E-stained images of thyroid glands from WT (^+/+^) and *TG^rdw/rdw^* rats (*n =* 6 per group), showing abnormally heterogeneous eosinophilic content in the follicle lumen, surrounded by abnormally swollen thyrocytes in *TG^rdw/rdw^* rats. Scale bars: 20 μm. (**B**) Representative distribution of aminopeptidase N by immunofluorescence (green) with DAPI counterstain (blue) in thyroid follicles of WT and *TG^rdw/rdw^* rats (*n =* 4 per group). For clarity, a yellow dotted line highlights the outer boundary of the thyroid follicular cells. Scale bars: 10 μm. (**C**) Representative distribution of BiP by immunofluorescence (red) with DAPI counterstain (blue) in thyroid follicles of WT and *TG^rdw/rdw^* rats (*n =* 4 per group). Scale bars: 10 μm. (**D**) Representative thyroid homogenates from WT and *TG^rdw/rdw^* rats were either mock digested or digested with endoglycosidase H (Endo H), followed by SDS-PAGE and immunoblotting with anti-Tg (*n =* 4 animals per group). No Tg from *TG^rdw/rdw^* rats was endo H resistant. (**E**) Representative distribution of Tg in the thyroid follicle lumen by immunofluorescence (green) with DAPI counterstain (blue) from WT and *TG^rdw/rdw^* rats (*n =* 7 per group). Scale bars: 20 μm.

The adult homozygous *TG^rdw/rdw^* rat (encoding Tg-G2298R) is well-known for congenital hypothyroidism, although rather than goiter, the animal develops a hypoplastic thyroid gland (36, 60–62). As in other vertebrates, the normal rat thyroid exhibits a classic monolayer of epithelial thyrocytes surrounding a central cavity filled with secreted eosinophilic Tg (Figure 3A, left). *TG^rdw/rdw^* rat thyroid glands also form follicles surrounding a central cavity with eosinophilic content (Figure 3A, right). Although suitable antibodies were not available to confirm the apical distribution of (rat) thyroid peroxidase and DUOX2 (two enzymes that help to catalyze T_4_ synthesis), we could confirm that aminopeptidase-N — also known to be an apical membrane marker in thyrocytes (63, 64) — was still delivered to its correct destination in *TG^rdw/rdw^* thyroid follicles (Figure 3B, a dashed yellow line highlights the basal membrane outlining the outer boundary of thyroid follicles). Nevertheless, the *TG^rdw/rdw^* thyroid histology was far from normal — the cytoplasm was massively engorged with eosinophilic vacuoles displacing nuclei under the apical plasmalemma, and the staining of the follicle lumen was abnormally heterogeneous (Figure 3A, right). The eosinophilic vacuoles are in fact ER (60) filled with the ER molecular chaperone, BiP (Figure 3C). Whereas more than 85% of Tg molecules in WT rats were Endo H resistant, analysis of *TG^rdw/rdw^* thyroid glands (*n =* 4) showed that the fraction of Tg molecules bearing Endo H resistance was 0, indicating an inability of mutant Tg to undergo intracellular transport to the Golgi complex (e.g., Figure 3D), as previously reported (65). Nevertheless, 64.5% ± 18.5% (SD, *n =* 6 animals) of *TG^rdw/rdw^* thyroid follicles exhibited mutant Tg in the lumen — associated with whole-cell profiles (Figure 3E) that upon close histological inspection indicated the presence of thyrocytes at different stages of cell death (Supplemental Figure 3A).

**Figure 4:**
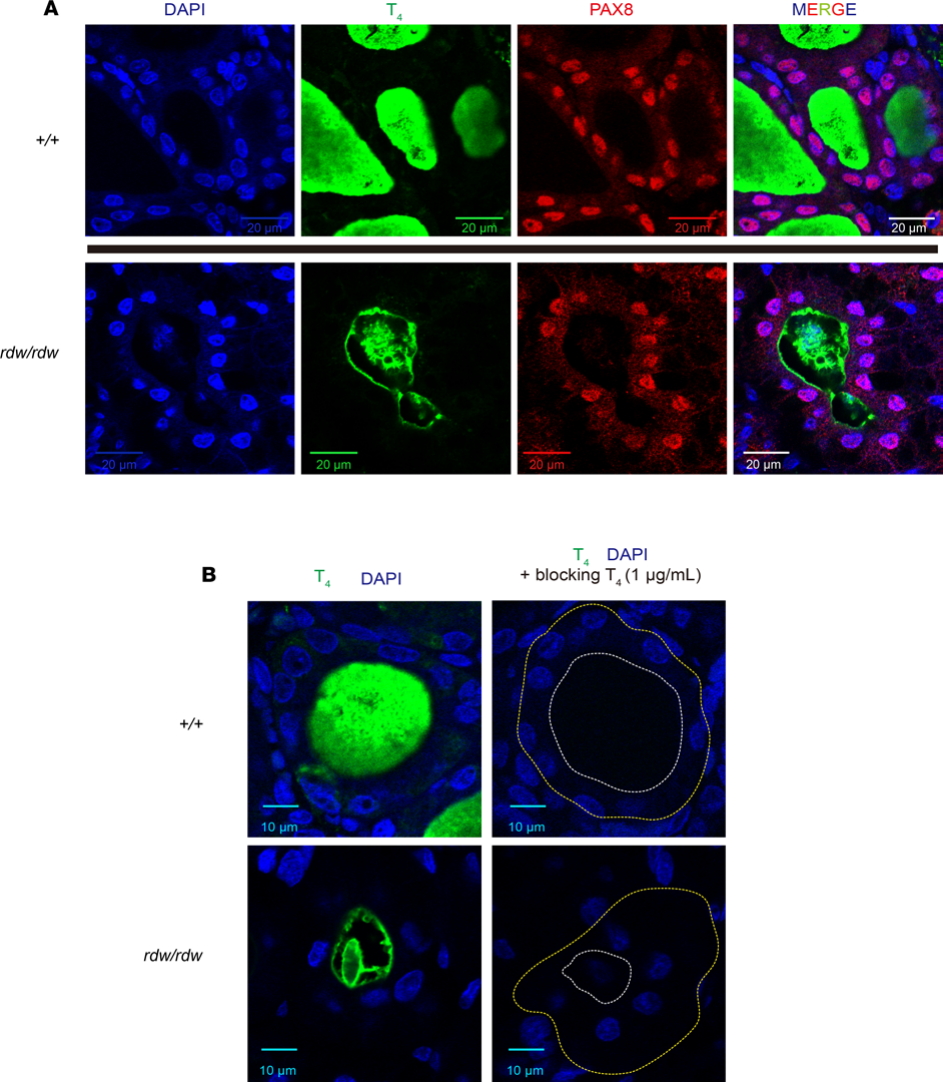
T4 synthesis in *TG^rdw/rdw^* rats. (**A**) Representative immunofluorescence of T4-containing protein (green) in the thyroid follicle lumen of WT (^+/+^) and *TG^rdw/rdw^* rats (*n =* 5 animals per group). Thyrocyte identity is confirmed by PAX8-positive nuclei (red) with DAPI counterstain (blue). Scale bars: 20 μm. (**B**) Representative immunofluorescence detection of T4-containing protein (green; with DAPI counterstain in blue) in the thyroids of WT and *TG^rdw/rdw^* rats (*n =* 9 animals per group) was specifically blocked by addition of T4 competitor (1 μg/mL). For clarity, a dashed white line delimits the thyroid follicle lumen; a yellow dotted line highlights the outer boundary of the thyroid follicle. Scale bars: 10 μm.

As in humans and mice with congenital hypothyroidism with mutant Tg, T_4_-containing protein — while dramatically diminished relative to that found in WT thyroid follicles — was apparent in the lumen of 89.7% ± 13.3% (SD, *n =* 5 animals) of the thyroid follicles of untreated *TG^rdw/rdw^* rats (Figure 4), surrounded by a monolayer of Pax8-positive cells (indicative of thyrocytes, Figure 4A). This T_4_-containing protein signal was specific, as it (a) could not be detected in the parotid salivary gland (that expresses sodium-iodide symporter but cannot iodinate proteins, Supplemental Figure 3B); (b) was fully blocked when adding soluble levoT_4_ competitor during the immunofluorescence protocol (Figure 4B, a dashed white line highlights the apical luminal cavity and a dashed yellow line highlights the basal membrane outlining the outer follicle boundary); and (c) was diminished in the thyroids of *TG^rdw/rdw^* rats fed chow containing propylthiouracil (which inhibits thyroid iodination, Supplemental Figure 3C). We could not continue the experiment to complete depletion of thyroidal T_4_-containing protein because within 4 weeks of treatment with the antithyroid drug, the animals became moribund and died spontaneously, indicating that ongoing endogenous T_4_ synthesis is required to avoid postnatal lethality.

**Figure 5:**
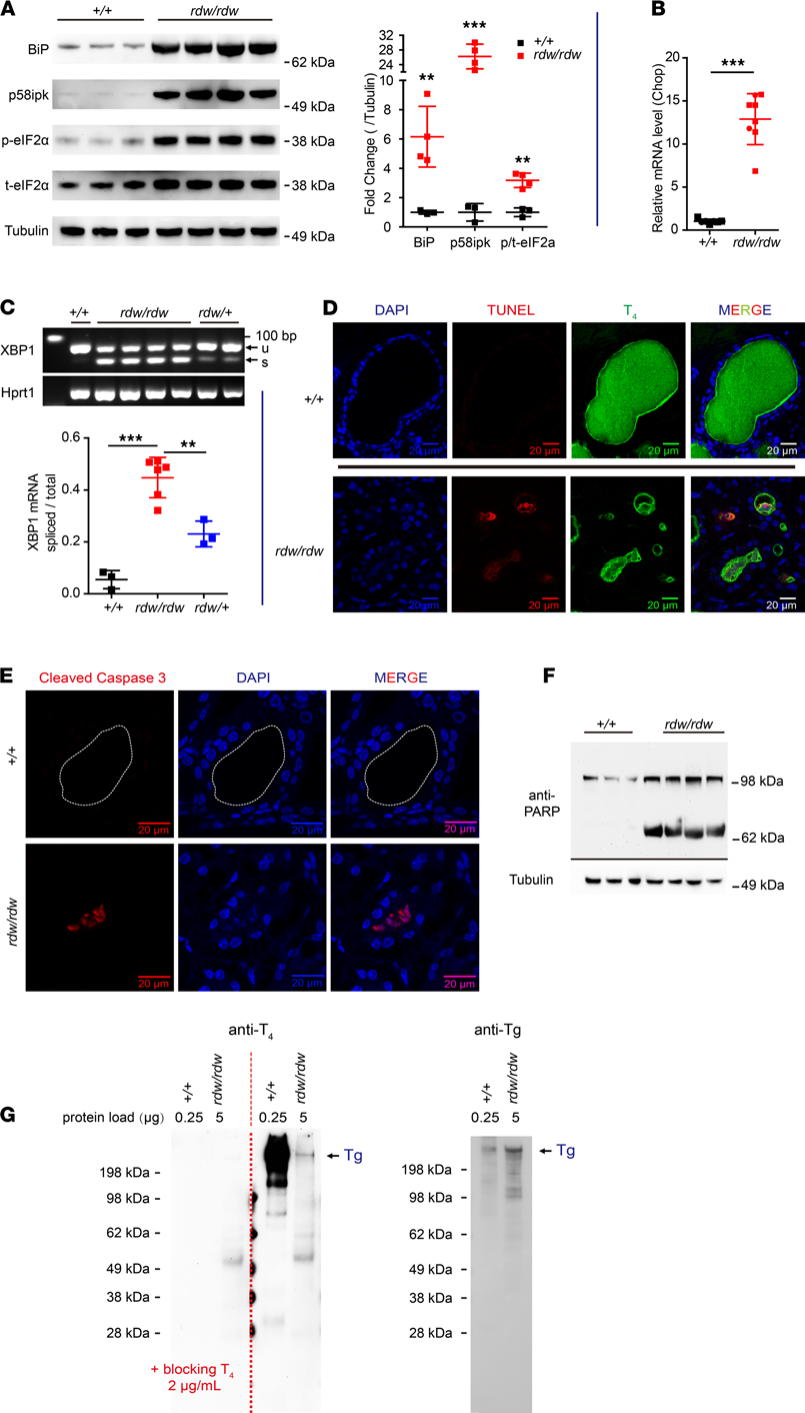
ER stress, cell death, and T4 synthesis in *TG^rdw/rdw^* rats. (**A**) Left: BiP, p58ipk, and phospho-eIF2α Western blotting in thyroids of WT (^+/+^) and *TG^rdw/rdw^* rats (each lane represent 1 animal). Right: Quantification (BiP and p58ipk normalized to tubulin; phospho-eIF2α normalized to total eIF2α; mean ± SD). **P < 0.01, ***P < 0.001 (unpaired 2-tailed Student’s *t* test). (**B**) CHOP mRNA levels (normalized to YWHAZ) in the thyroid glands of WT and *TG^rdw/rdw^* rats (*n =* 7–8 animals/group; each point represents 1 animal; mean ± SD). ***P < 0.001 (unpaired 2-tailed Student’s *t* test). (**C**) Top: Representative samples showing spliced and unspliced XBP1 mRNA in the thyroids of WT, *TG^rdw/+^*, and *TG^rdw/rdw^* rats (*n =* 3–6 animals/group; each lane represents 1 animal). Hprt1 was used as a loading control. Bottom: Quantitation of the fraction of spliced XBP1 (mean ± SD). **P < 0.01, ***P < 0.001 (1-way ANOVA, Bonferroni post hoc test). (**D**) Representative TUNEL staining and immunofluorescence of T4-containing protein with DAPI counterstain in the thyroids of WT and *TG^rdw/rdw^* rats (*n =* 4 animals/group). Scale bars: 20 μm. (**E**) Representative immunofluorescence of cleaved caspase-3 with DAPI counterstain in thyroids of WT and *TG^rdw/rdw^* rats (*n =* 5 animals/group). For clarity, a dashed white line delimits the thyroid follicle lumen in the WT rats (in which cleaved caspase-3 is not detectable). Scale bars: 20 μm. (**F**) Western blotting of PARP in thyroid glands from WT and *TG^rdw/rdw^* rats (*n =* 3–4; each lane represents 1 animal). (**G**) Left: Representative Western blotting of T4-containing protein in thyroid homogenates of WT and *TG^rdw/rdw^* rats (*n =* 5 animals/group) with or without soluble competitor T4 to block specific bands (left of dotted red line). Right: The same samples immunoblotted with mAb anti-Tg showing intentional overloading of the *TG^rdw/rdw^* rat sample.

We examined ER stress responses in *TG^rdw/rdw^* thyroid glands. PERK phosphorylation of eIF2α stimulates increased translation of ATF4 that upregulates CHOP, which (as noted above) has been strongly implicated in cell death (29). In addition to a dramatic increase of BiP and p58ipk, *TG^rdw/rdw^* thyroid glands were observed to have increased phosphorylated eIF2α (Figure 5A), accompanied by a more than 10-fold increase of CHOP mRNA (Figure 5B). A second ER stress–related death pathway involves IRE1 hyperactivation that can trigger a “terminal UPR” from exuberant RNAse activity (“RIDD”), which typically develops only in cells exhibiting demonstrably high levels of stress-induced IRE1 splicing of XBP1 mRNA (66). We observed that roughly half of thyroidal XBP1 mRNA was spliced to the active form in *TG^rdw/rdw^* animals (Figure 5C), which is impressive considering that thyrocytes and C cells together constitute only approximately 60% of resident cells in the mouse thyroid (67); i.e., these are conditions that can favor a terminal UPR. Moreover, TUNEL-positive cells were present in the follicular lumina of *TG^rdw/rdw^* thyroid glands. Indeed, the thyroid follicles of untreated *TG^rdw/rdw^* rats exhibited ongoing T_4_ synthesis (Figure 5D), with 27.2% ± 5.3% (SD, *n =* 3 animals) of all follicles triply positive for weak DAPI staining, TUNEL, and T_4_-containing protein; a positive signal for cleaved caspase-3 was also detected in 24.8% ± 9.4% of thyroid follicles (SD, *n =* 5 animals), although none of these features were detected in WT thyroid (Figure 5E). Caspase activity can cleave the DNA repair enzyme poly(ADP-ribose) polymerase (PARP), and unlike in WT thyroid tissue, PARP was extensively cleaved in *TG^rdw/rdw^* thyroid glands (Figure 5F).

We performed Western blotting of total thyroidal proteins with anti-T_4_. Despite the presence of background bands, an approximately 330 kDa band comigrating with WT Tg was the clearest T_4_-containing protein specifically identified in the thyroid tissue of untreated *TG^rdw/rdw^* rats. The intensity of this band was completely eliminated by the addition of free T_4_ competitor to the antibody incubation during Western blotting (Figure 5G, left). The efficiency of T_4_ formation in this protein indicative of mutant Tg was much less than in the Tg protein from WT rat thyroid glands, especially when considering that more Tg protein was loaded for the mutant sample (Figure 5G, right). Altogether, the data in Figures 3–5 support that *TG^rdw/rdw^* rats also use dead thyrocytes for endogenous T_4_ synthesis on mutant Tg.

**Figure 6:**
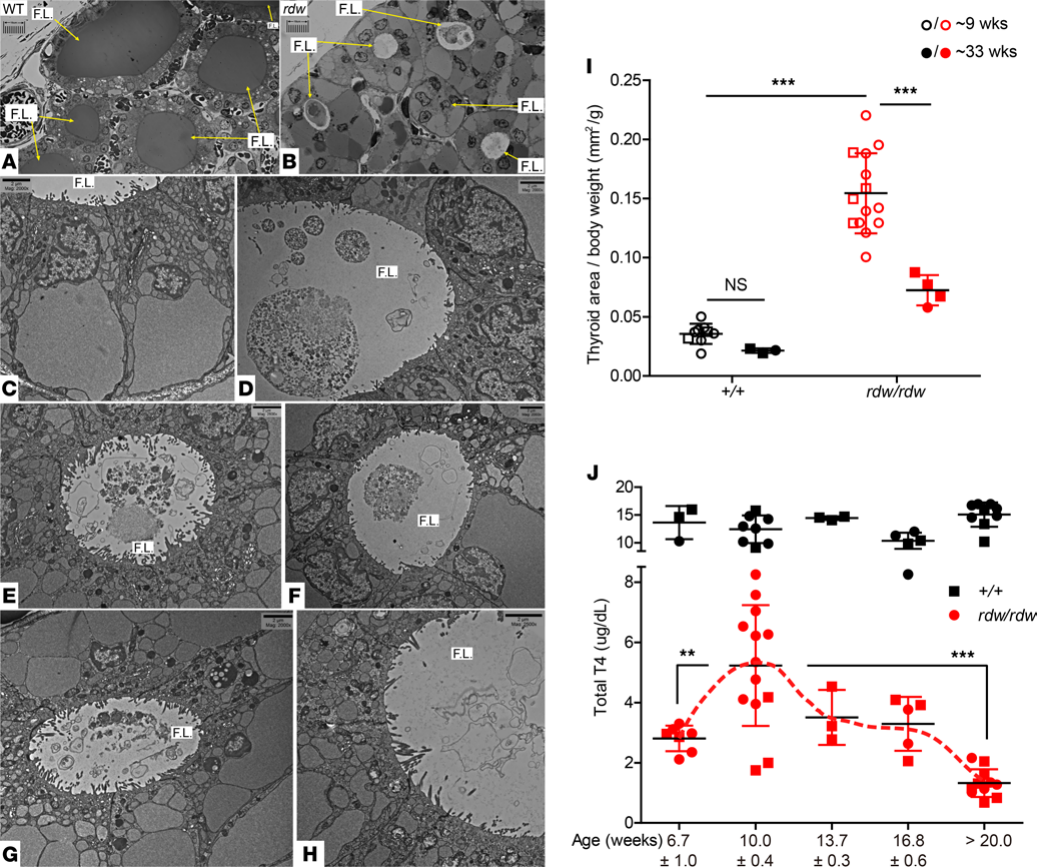
In congenital goiter with mutant TG, thyrocyte cell mass provides the dead-cell–derived substrate for T4 synthesis. (**A**–**H**) Microscopy of WT and *TG^rdw/rdw^* rat thyroid follicles. (**A**) WT rat thyroid. Cross-sections of several thyroid follicles are shown; each follicle lumen (F.L.) is acellular but filled with WT Tg protein (thin yellow arrows). Scale bar: 10 μm in 1.0 μm increments. (**B**) *TG^rdw/rdw^* rat thyroid. Yellow arrows point to the follicle lumina; note the enlarged cytoplasm and abnormal, cellular contents of the follicle lumina. Scale bar: 10 μm in 1.0 μm increments. (**C**–**H**) Transmission EM survey of *TG^rdw/rdw^* rat thyroid follicles. Scale bars: 2 μm. (**C**) Engorged ER vacuoles in the basal cytoplasm with apically displaced nuclei. (**D**–**H**) Dead-cell ghosts in various thyroid follicles, each at a different stage of cellular disintegration within the follicle lumen. (**G**) Living thyrocytes with abundant apical microvilli, which have internalized material from the follicle lumen into endo-lysosomes. (**H**) Until new dead cells enter the follicle lumen, there is progressive clearance of cellular debris from the luminal cavity. (**I**) Thyroid gland size (normalized to body weight) in a cohort of young versus older animals (open symbols represent rats at 8.9 ± 1.7 weeks of age; closed symbols represent rats at 33.4 ± 2.6 weeks of age; males are shown as squares and females as circles) (mean ± SD). ***P < 0.001 (2-way ANOVA, Bonferroni post hoc test). (**J**) Total T4 levels in serum of WT (^+/+^) and *TG^rdw/rdw^* rats as a function of age (males are shown as squares and females as circles) (mean ± SD). **P < 0.01, ***P < 0.001 (2-way ANOVA, Bonferroni post hoc test).

To more clearly examine the disintegration of dead thyrocytes bearing mutant Tg, fixed and post-fixed WT and *TG^rdw/rdw^* thyroid tissue were embedded in plastic for semithin sectioning. As expected, WT thyroid revealed dense, uniformly stained colloid (Tg protein) in the follicle lumen (Figure 6A). In contrast, in the thyroids of *TG^rdw/rdw^* rats, in addition to large vacuoles in the basal cytoplasm with apically displaced nuclei, the lumen of different follicles varied, with contents ranging from whole cells to cellular debris (Figure 6B). Moreover, electron microscopy revealed that living follicular thyrocytes had massively swollen ER with unusual nuclear morphology, and most of the remaining organelles were crowded into the apical cytoplasm, ultimately limited by the apical plasma membrane bearing microvilli that extend into the follicle lumen (Figure 6C). Cell ghosts with disintegrating organelles were readily apparent in many of the follicle lumina examined (e.g., Figure 6D), surrounded by epithelial cells bearing dense endo-lysosomes (Figure 6, E and F), suggesting that dead-cell material from the follicle lumen enters surrounding living thyrocytes via endocytic internalization, with progressive clearance of the detritus of dead-cell ghosts (Figure 6, G and H).

A great puzzle in the field has been understanding why some patients (and some animal models) with biallelic *TG* mutations that grow a large goiter can yield a survivable serum T_4_ level without treatment, yet other patients and animals models with an intact hypothalamic-pituitary-thyroid axis are unable to do so (68). With this question specifically in mind, we examined thyrocyte proliferation in hypothyroid *TG^rdw/rdw^* rats. Indeed, in early life we observed that *TG^rdw/rdw^* rats did indeed exhibit active proliferation of thyrocytes, similar to that observed in *TG^cog/cog^* mice (Supplemental Figure 4A). Indeed, although never previously described, we observed that in early life *TG^rdw/rdw^* rats do in fact develop thyroid enlargement (i.e., goiter) by 9 weeks of age (Figure 6I), and this parallels a significant increase of endogenous T_4_ synthesis that supports serum T_4_ levels (Figure 6J). However, as the *TG^rdw/rdw^* animals aged, the enlarged thyroid gland size could not be sustained (Figure 6I), and with this (61), the animals could not maintain their serum T_4_ levels (Figure 6J). Untreated profound hypothyroidism is ultimately incompatible with life in rodents (noted above, and refs. 69, 70) as well as in humans. It thus appears that only patients and animal models that can support a sufficient goiter are able to provide the continuous supply of dead thyrocytes needed for ongoing T_4_ synthesis — a mechanism that can allow some individuals the chance to sustain endogenous thyroid hormone levels in adulthood (24).

## Discussion

Reports describe untreated adult patients with a large goiter who are biochemically and clinically nearly euthyroid despite biallelic *TG* deficiency (71–73). Two longstanding but competing schools of thought are that (a) a large hyperplastic goiter is a compensatory physiological adaptation in response to thyroidal genetic or environmental factors that disfavor thyroid hormone production (74) or (b) growth of a large thyroid goiter is actually a maladaptation (28). On the one hand, because the Tg protein is the evolutionarily preferred thyroid hormone precursor (1), and because the mutant *TG* alleles encode a Tg protein that cannot be exported via the secretory pathway from the ER to the site of iodination (2, 10), simply enlarging the thyroid gland does seem pointless. On the other hand, in this disease, there are reasons favoring a positive correlation between thyroid follicular cell mass and the overall synthesis of T_4_ needed to sustain serum T_4_ levels (41).

The main finding of this report is that, in humans and animals with biallelic mutant *TG*, T_4_ biosynthesis continues as long as the thyroid follicle lumen is provided with a supply of dead or dying thyrocytes (see schematic cartoon, Supplemental Figure 4B). Throughout the course of the disease, a fraction of the ER-stressed thyrocytes die and are extruded to the luminal cavity, temporarily including activation of caspase-3, cleavage of PARP, and DNA cleavage leading to nuclear fragmentation and karyolysis, ultimately leading to complete disintegration of cellular organelles within the iodination environment of the follicle lumen. Death of thyrocytes is asynchronous, chronic, and heterogenous during the course of the disease, such that at any moment in time, cells at various stages of demise are observed across the gland, surrounded by living cells that maintain a follicular architecture enclosing iodoproteins within the apical cavity. Our analysis did not include specific detection of inflammatory cell infiltration of thyroid follicles but rather ER stress throughout the entire population of living mutant thyrocytes, with remnant detection of ER stress (e.g., CHOP) in the dead-cell ghosts. Crucially, in this disease, it is the dead, disintegrating thyrocytes upon which T_4_-containing protein can be detected, with the thyroid follicles cannibalizing (internalizing) the iodinated detritus of dead thyrocytes into the surrounding living follicular cells.

The spectrum of proteins upon which T_4_ might be made inefficiently in humans with biallelic *TG* mutation has not yet been fully explored, although it has often been speculated that albumin, which can become highly iodinated (75) as a serum protein capable of transcytosis (76) or paracelllular leakage (75), could perhaps be a source of endogenous T_4_. Given the mechanistic understanding presented in the current study, we recognize that the entire proteome leaked from dead thyrocytes becomes eventually exposed to the iodination environment. Here, we show that even though the mutant Tg protein cannot be secreted via conventional intracellular trafficking (77), it is nevertheless conveyed to the lumen of thyroid follicles via dead thyrocytes, wherein T_4_ is produced (albeit inefficiently) within the mutant Tg protein in *TG^cog/cog^* mice, *TG^rdw/rdw^* rats, and, most likely, humans with the same disease.

Indeed, this pathological salvage mechanism of T_4_ synthesis is observed in the goitrous thyroid gland of a patient with homozygous expression of Tg-W2346R. Moreover, in this study, we provide strong supporting evidence that total thyroid cell mass (i.e., the goiter) is important for the endogenous rescue from hypothyroidism. Specifically, increasing amounts of T_4_ are produced as the thyroid begins to grow postnatally (41); however, in *TG^rdw/rdw^* rats, profound hypothyroidism ensues in parallel with an atrophic thyroid gland (36, 61). Thus, our results demonstrate that *TG^rdw/rdw^* rats form a goiter but cannot sustain their goiter with aging. Evidently, without the growing goiter, the thyroid gland cannot provide sufficient dead cells needed to fuel ongoing thyroid hormone production. These considerations make it clear that the balance of cell proliferation-versus-death is indeed a critical factor, as ultimately, an unfavorable balance in *TG^rdw/rdw^* rats deprives the thyroid of sufficient substrate to maintain T_4_ synthesis. In contrast, continued growth of the goiter in *TG^cog/cog^* mice allows this pathological salvage mechanism to endogenously self-correct the hypothyroidism (41), and human studies suggest a similar conclusion in patients with a goiter with biallelic *TG* mutations.

More work is needed to determine if *rdw*Tg might somehow be more proteotoxic than *cog*Tg (40) or if other factors that vary between species drive the enhanced capability of *TG^cog/cog^* mice for a net proliferation of thyrocytes (i.e., in excess of cell death) into adulthood. What is apparent in all cases, however, is that the continuous contribution of dead and dying thyrocytes, which provide substrate for T_4_ production, represents the critical compensatory response to congenital hypothyroidism with biallelic *TG* mutations. Moreover, as a brief exposure to antithyroid drug is lethal to *TG^rdw/rdw^* rats, our findings appear consistent with the hypothesis that in the presence of biallelic *TG* mutations, survival of the organism (8, 68) does require this most unusual means of thyroid hormonogenesis.

## Methods

### Primary antibodies.

Anti-Ki67 (SP6) (ab16667, Abcam); anti–cleaved caspase-3 (Asp175) antibody (9661, Cell Signaling); anti-CHOP (sc-7351, Santa Cruz); anti-T_4_ (1H1) (sc-52247, Santa Cruz); mAb anti-Tg (365997, Santa Cruz; ab156008, Abcam); rabbit anti-Tg and rabbit anti-BiP were previously described (38, 45); rabbit anti-Pax8 (10336-1-AP, ProteinTech Group); mAb anti-Aminopeptidase N (1D7) was the gift of D. Fox, University of Michigan (78); rabbit anti-p58ipk (2940, Cell Signaling Technology); rabbit anti-phospho-eIF2α (Ser51) (3597, 9721, Cell Signaling) and total eIF2α (9722, Cell Signaling); mouse anti-tubulin (T5168, MilliporeSigma); and rabbit anti-PARP (9542, Cell Signaling).

### Human thyroid sections.

A homozygous patient bearing the *TG*-W2346R mutation was previously described (including parental written informed consent and appropriate IRB approval, ref. 15). Paraffin blocks of deidentified surgically resected thyroid tissue, including large regions of normal human thyroid from 3 now-deceased patients, were obtained from the Molecular Pathology Research Laboratory, University of Michigan, and were sectioned and H&E stained or processed for immunofluorescence as described below.

### Animals.

*TG^cog/cog^* mice (C57BL/6) were from JAX. *TG^rdw/+^* rat heterozygotes were obtained from the National BioResource Project in Japan (NBRP rat no. 0104) and bred to homozygosity; WT animals were littermates of the same strain background. Adult 2- to 4-month-old mice were used in all studies, except in Figure 1G, Supplemental Figure 1C, and Supplemental Figure 4 (in which 9- to 16-month-old mice with larger goiter were used for better yield of T_4_-containing proteins and Ki67-positive thyrocytes). Four- to eighteen-week-old rats were used in all studies (i.e., after weaning), except in Figure 6, I and J, in which detailed ages are shown. In all figures, data from male and female animals were combined, except in Figure 6, I and J, in which results from each individual animal are shown.

### Cell culture.

PCCL3 cells (79) (B. DiJeso, University of Salento, Lecce, Italy) were maintained in DMEM/F-12 with 5% fetal bovine serum, 1 mIU/mL thyrotropin, 1 μg/mL insulin, 5 μg/mL apo-transferrin, 1 nM hydrocortisone (4 hormones obtained from MilliporeSigma), and penicillin/streptomycin. PCCL3-conditioned media containing secreted TG were collected as a positive control for Endo H–resistant TG.

### Thyroid gland size measurement.

Thyroids of euthanized animals were dissected, with both lobes of the gland fully exposed. Images of the neck were captured with a calibrated size marker included in situ. The areas of the thyroid glands (correlated with volume) were measured using ImageJ (NIH) and quantified as a fraction of body weight of each animal.

### Serum total T_4_ measurement.

Whole blood was collected, clotted, and centrifuged at 750*g* for 20 minutes to obtain serum. Total T_4_ was assayed by ELISA (Diagnostic Automation/Cortez Diagnostics).

### Preparation and immunostaining of thyroid sections.

Thyroid glands from mice and rats were immersion-fixed with 10% formalin and processed for paraffin embedding, sectioning, and H&E staining. For immunofluorescence, 6 μm sections were deparaffinized in Citrisolv and an ethanol series, then heated in citrate buffer (12.3 mM, pH 6) for antigen retrieval, and blocked in 1.5% normal goat serum for 30 minutes at room temperature. Primary antibody incubation was performed overnight at 4°C, followed by incubation of Alexa Fluor–conjugated secondary antibodies (Thermo Fisher). After washing, sections were counterstained and mounted with Prolong-Gold and DAPI (Invitrogen). Images were captured in a Nikon A1 confocal microscope. For anti-Ki67 immunohistochemistry, the VECTASTAIN ABC Kit (Vector) was used. After antigen retrieval, sections were treated with 3% H_2_O_2_, blocked in 1.5% normal goat serum for 20 minutes at room temperature, and incubated with anti-Ki67 antibody for 1 hour at room temperature and biotinylated secondary antibody for 30 minutes, followed by incubation with avidin-HRP. Staining was visualized by DAB reaction. Sections were counterstained with hematoxylin, dehydrated in a graded series of ethanol, and mounted with Permount. Images were obtained with a Leica DMI-3000B microscope.

### T_4_ immunofluorescence/TUNEL double labeling.

The ApopTag In Situ Apoptosis Detection Kit (Millipore) was used for TUNEL staining of thyroid sections. Minor modifications were applied for double immunofluorescence labeling with anti-T_4_. Briefly, deparaffinized thyroid tissue sections were pretreated with proteinase K (20 μg/mL) and blocked in 1.5% normal goat serum for 30 minutes at room temperature. Incubation with anti-T_4_ antibody was performed at room temperature for 1 hour, followed by incubation with Alexa Fluor 488–conjugated secondary antibody for 30 minutes at room temperature. After washing, TUNEL staining was performed. Sections were counterstained and mounted with Prolong-Gold and DAPI (Invitrogen). Fluorescence images were captured in a Nikon A1 confocal microscope.

### Endo H digest.

Thyroid homogenates from WT or mutant mice and rats were boiled in denaturing buffer containing 0.5% SDS and 40 mM dithiothreitol (DTT) at 95°C for 5 minutes, cooled, and then either mock digested or digested with Endo H (1000 units, NEB) for 1 hour at 37°C.

### Western blotting.

Rodent thyroid glands were homogenized in RIPA buffer (25 mM Tris•HCl pH 7.6, 150 mM NaCl, 1% NP-40, 1% sodium deoxycholate, 0.1% SDS, Thermo Fisher Scientific), including either protease inhibitor cocktail (Roche) or protease-plus-phosphatase inhibitor cocktail (Thermo Fisher Scientific). Total protein concentration was determined by BCA assay (Thermo Fisher Scientific). Lysates were boiled in SDS-gel sample buffer with 50 mM DTT. Samples were then resolved by SDS 4.5%-PAGE or SDS 4%–12%-PAGE, electrotransferred to nitrocellulose, and blocked with 5% milk before immunoblotting with the indicated antibodies and appropriate HRP-conjugated secondary antibody, and visualized by enhanced chemiluminescence. Band quantitation was performed using ImageJ (NIH).

### Immunoprecipitation analysis of T_4_-containing protein.

Mouse thyroid glands were homogenized in RIPA buffer plus protease inhibitor cocktail (Roche). Thyroid homogenates were incubated with mAb anti-T_4_ antibody and protein G-agarose (Exalpha Biologicals) overnight at 4°C. Precipitates were washed 3 times in RIPA buffer (and for samples to be digested with Endo H, 1 additional washes in PBS) and then boiled in SDS gel–sample buffer containing 50 mM DTT, resolved by SDS 4.5%-PAGE or SDS 4%–12%-PAGE, electrotransferred to nitrocellulose, and immunoblotted with anti-T_4_ or anti-Tg antibody.

### PCR.

Total RNA was purified from the thyroid gland tissue or PCCl3 cells using a RNeasy Plus kit (Qiagen). Synthesis of cDNA was performed using SuperScript III First-Strand Synthesis SuperMix (Invitrogen) or High-Capacity cDNA Reverse Transcription Kits (Thermo Fisher Scientific). For XBP1 splicing analysis, the primers below were designed to encompass the IRE1 cleavage site of XBP1: forward primer, 5′-TGGCCGGGTCTGCTGAGTCCG-3′, and reverse primer, 5′-ATCCATGGGAAGATGTTCTGG-3′. The amplicons, including the spliced (71 bp) and unspliced (97 bp) XBP1, were generated using the GoTaq Green Master Mix Kit (Promega). PCR products were resolved by a 3% agarose gel. Hypoxanthine phosphoribosyltransferase 1 (Hprt1) was used as a control, amplified using the following primers, and loaded as a loading control: forward primer, 5′-CTCATGGACTGATTATGGACAGGA-3′, and reverse primer, 5′-GCAGGTCAGCAAAGAACTTATAGCC-3′. Band quantitation was performed using ImageJ (NIH). For real-time PCR, TaqMan Universal Master Mix was used on a StepOnePlus PCR system (Thermo Fisher Scientific). CHOP gene expression was normalized to that of Ywhaz; both probes were from TaqMan Gene Expression Assays (Thermo Fisher Scientific).

### Electron microscopy of rat thyroid glands.

WT and *rdw/rdw* rats were briefly perfusion fixed with HEPES-buffered 2% glutaraldehyde before thyroid dissection and continuing as immersion fixation. The tissues was then washed in 100 mM Na cacodylate containing 2 mM CaCl_2_, before postfixation with 0.25% OsO_4_, further washing, and staining with 0.5% uranyl acetate. After additional washes, the tissue was dehydrated in a graded series of ethanol (50%, 75%, 95%, and 100%) followed by a 30-minute incubation in propylene oxide. The tissue was finally infiltrated with Araldite in propylene oxide and then pure Araldite, which was polymerized under heating. 10 μm plastic sections were stained with 1% toluidine blue and examined by light microscopy (×100 objective); then 0.5 μm sections were picked up and placed on Formvar-coated copper grids. If not, edit for clarity, poststained with 1% lead citrate, and rinsed prior to examination in a JEOL-JEM-1400 transmission electron microscope.

### Statistics.

Statistical analyses were calculated using GraphPad Prism. Data are represented as mean ± SD. Unpaired 2-tailed Student’s *t* test was used for comparisons between 2 groups. One-way ANOVA with Bonferroni post hoc test was used for comparison of 3 groups. Thyroid size and serum T_4_ measurements were analyzed by 2-way ANOVA with Bonferroni post hoc test. Statistical significance was determined at *P <* 0.05.

### Study approval.

All experiments performed with mice and rats were in compliance with and approved by the University of Michigan Institutional Animal Care and Use Committee (PRO00009936). The patient thyroid block was obtained with parental written informed consent and IRB approval from the University of Buenos Aires (CEIC FFyB: 1704 2019-85; ref: EXP-FYB, no. 0067072/2018).

## Author contributions

XZ, CEC, YM, and PA designed the experiments. XZ, APK, and CEC performed. the experiments, with assistance provided by HZ and DL. HMT and VAB provided key reagents. wrote the Methods. PA supervised the work and wrote the manuscript. All authors reviewed, edited, and approved the manuscript.

## Supplementary Material

Supplemental data

## Version 1 04/29/2021

In-Press Preview

## Version 2 06/08/2021

Electronic publication
