# Supplementary material for: Thyroid hormone synthesis continues despite biallelic thyroglobulin mutation with cell death
Source: JCI Insight. 2021 Jun 8;6(11):e148496. doi: 10.1172/jci.insight.148496 (PMC8262357; doi:10.1172/jci.insight.148496)
Supplement: Supplemental data [file jciinsight-6-148496-s133.pdf]

Supplemental Figures

Fig. S1. Detection of CHOP and cleaved caspase-3 in thyroid follicles of $TG^{cog/cog}$ mice. **A**. CHOP immunofluorescence (red) with DAPI counter-stain (blue) in thyroid glands of WT and $TG^{cog/cog}$ mice (n=3); scale bars = 20 µm. Note karyolysis: chromatin expansion with abnormally diminished DAPI staining in the nuclei that are positive for CHOP. **B**. Cleaved caspase-3 immunofluorescence (red) with DAPI counter-stain (blue) in thyroid glands of WT and $TG^{cog/cog}$ mice (n=3-4) ; scale bars = 20 µm. **C**. Thyroid homogenates from WT ("B6") and three independent $TG^{cog/cog}$ mice (*cog* 1-3) were analyzed either before or after immunoprecipitation with mAb anti-T_4, followed by immunoblotting with either the same antibody (lanes 1-6) or with mAb anti-Tg the recognizes an epitope in the region of Tg residues 1000-1100. In the immunoblotting of lane 6 (blue asterisk), 2 µg/mL of competitor T_4 was added as a specificity control. In lane 8, identical immunoprecipitation and immunoblotting was performed, but in the absence of thyroid homogenate. The Tg protein bearing T_4 as shown in lanes 9 – 11 are derived from the initial samples shown in lanes 2 – 4, respectively. Two additional $TG^{cog/cog}$ mice (total n=5) run on a separate gel yielded the same results.

Fig. S2. Dead and dying thyrocytes in the thyroid follicular lumina in human goitrous hypothyroidism caused by homozygous $TG^{W2346R/W2346R}$. **A.** H&E images from a surgical specimen of the thyroid gland of the patient; scale bars = 10 µm. **B-D**. Representative images of TUNEL staining (red) and immunofluorescence of T_4-containing protein (green) with DAPI counter-stain (blue) in the thyroid gland of the same patient; scale bars = 20 µm. The immunofluorescence was repeated 3 times.

Fig. S3. Screening for T_4-containing protein in $TG^{rdw/rdw}$ rats. **A.** A sampling of thyroid follicles from H&E images of the thyroid glands of $TG^{rdw/rdw}$ rats (n=4) ; scale bars = 20 µm, showing dying and dead thyrocyte ghosts at different stages, in the follicle lumen. **B**. Low power immunofluorescence demonstrating specificity of T_4-containing protein (green; with DAPI counter-stain in blue) in the thyroid gland (*upper panels*) *versus* the parotid gland (*lower panels*) from a $TG^{rdw/rdw}$ rat (gland

architecture shown in insets at left, scale bars = 20 µm). The immunofluorescence was repeated 3 times ; scale bars = 50 µm. **C**. Low power immunofluorescence of T_4-containing protein (green; with DAPI counter-stain in blue) in thyroid glands of $TG^{rdw/rdw}$ rats (n=3-5) either untreated (*upper panels*) or treated with propylthiouracil chow (PTU for 27 d, *lower panels*) ; scale bars = 200 µm..

Fig. S4. Thyrocyte proliferation, and model of thyroxine synthesis in congenital hypothyroidism from mutant *TG*. **A**. Ki67 immunohistochemistry of the thyroid gland of WT (n=4) and $TG^{rdw/rdw}$ rats (n=6) as well as $TG^{cog/cog}$ mice (n=2); scale bars = 50 µm. There are essentially no Ki67-positive nuclei detectable in the WT control thyroid. **B**. Schematic cartoon of thyroid hormone synthesis under normal conditions, and in congenital goiter with bi-allelic *TG* mutation.

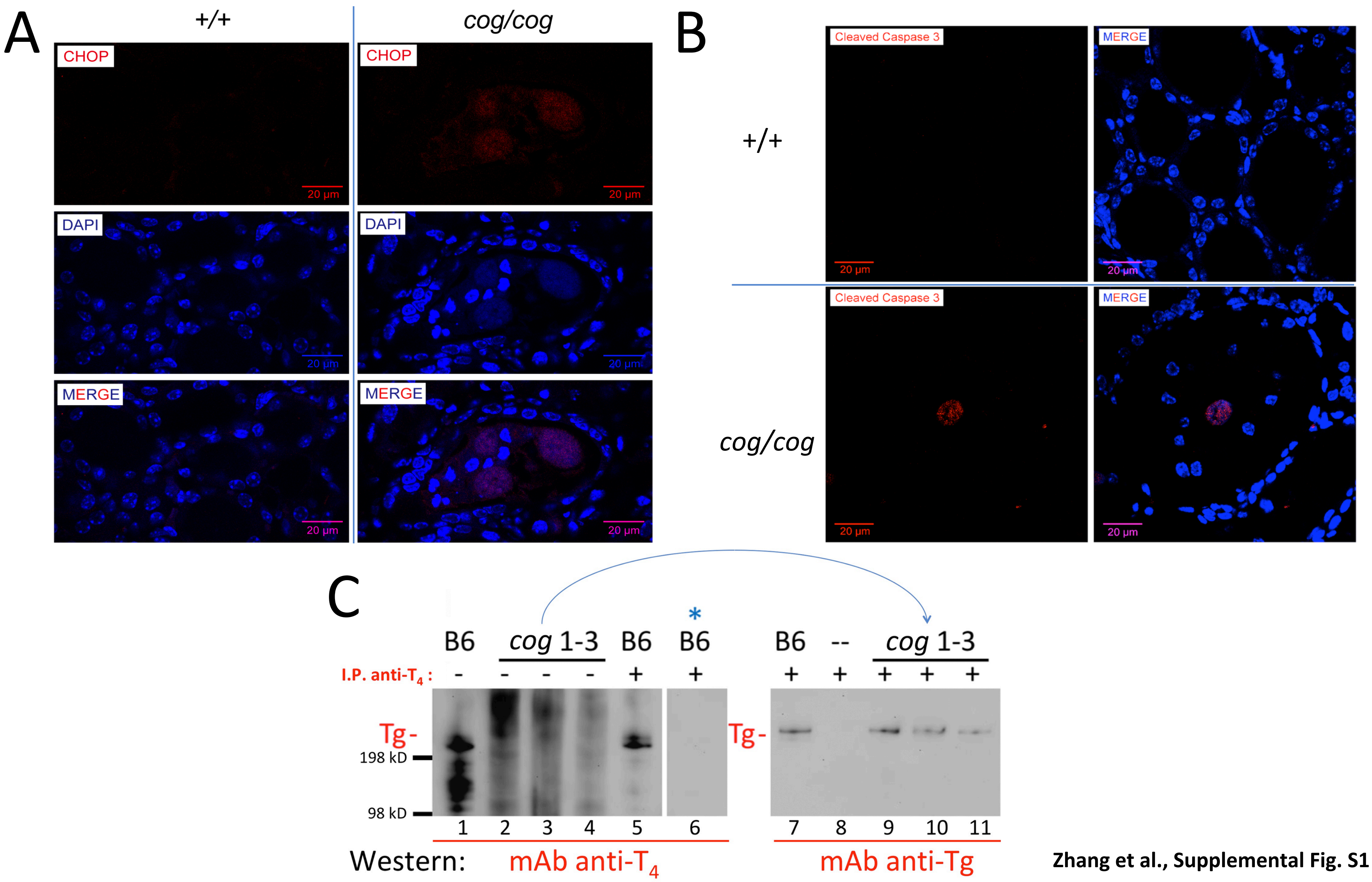

Zhang et al., Supplemental Fig. S1

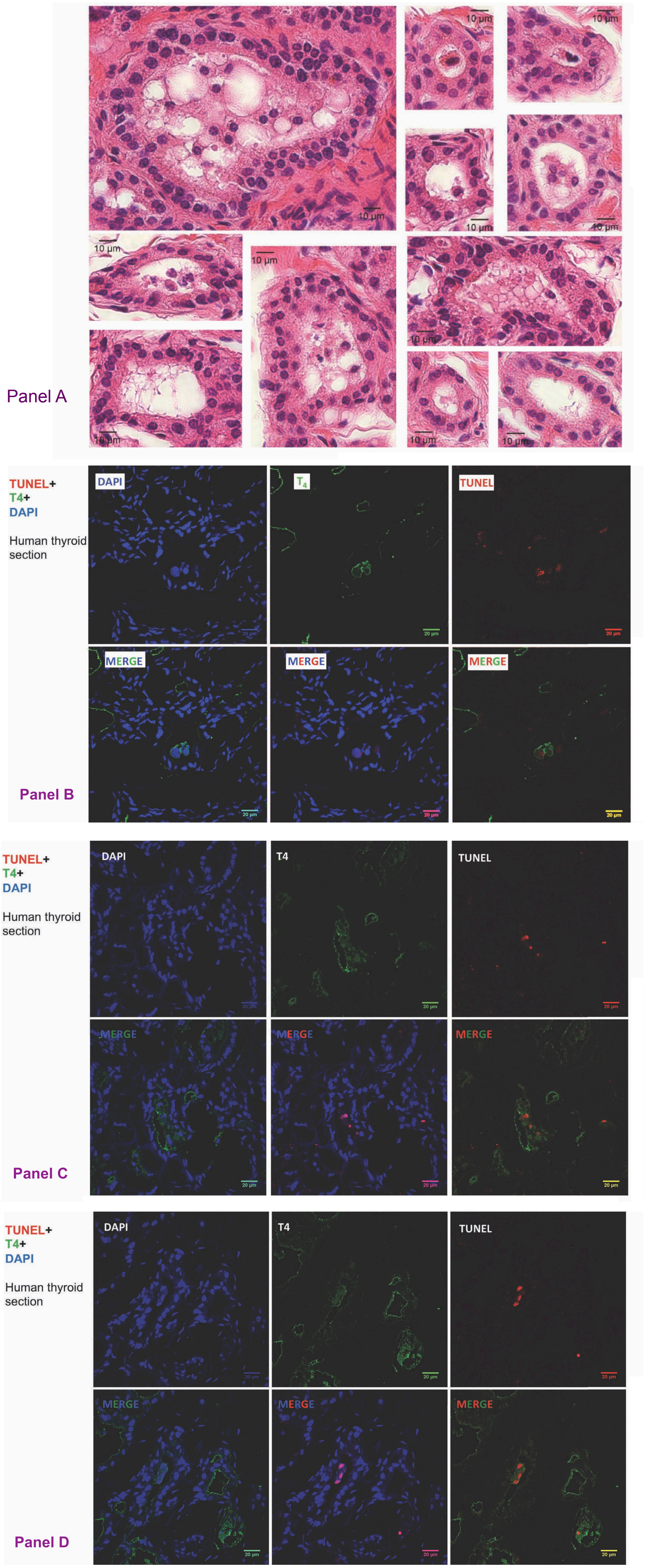

Zhang et al., Supplemental Fig. S2

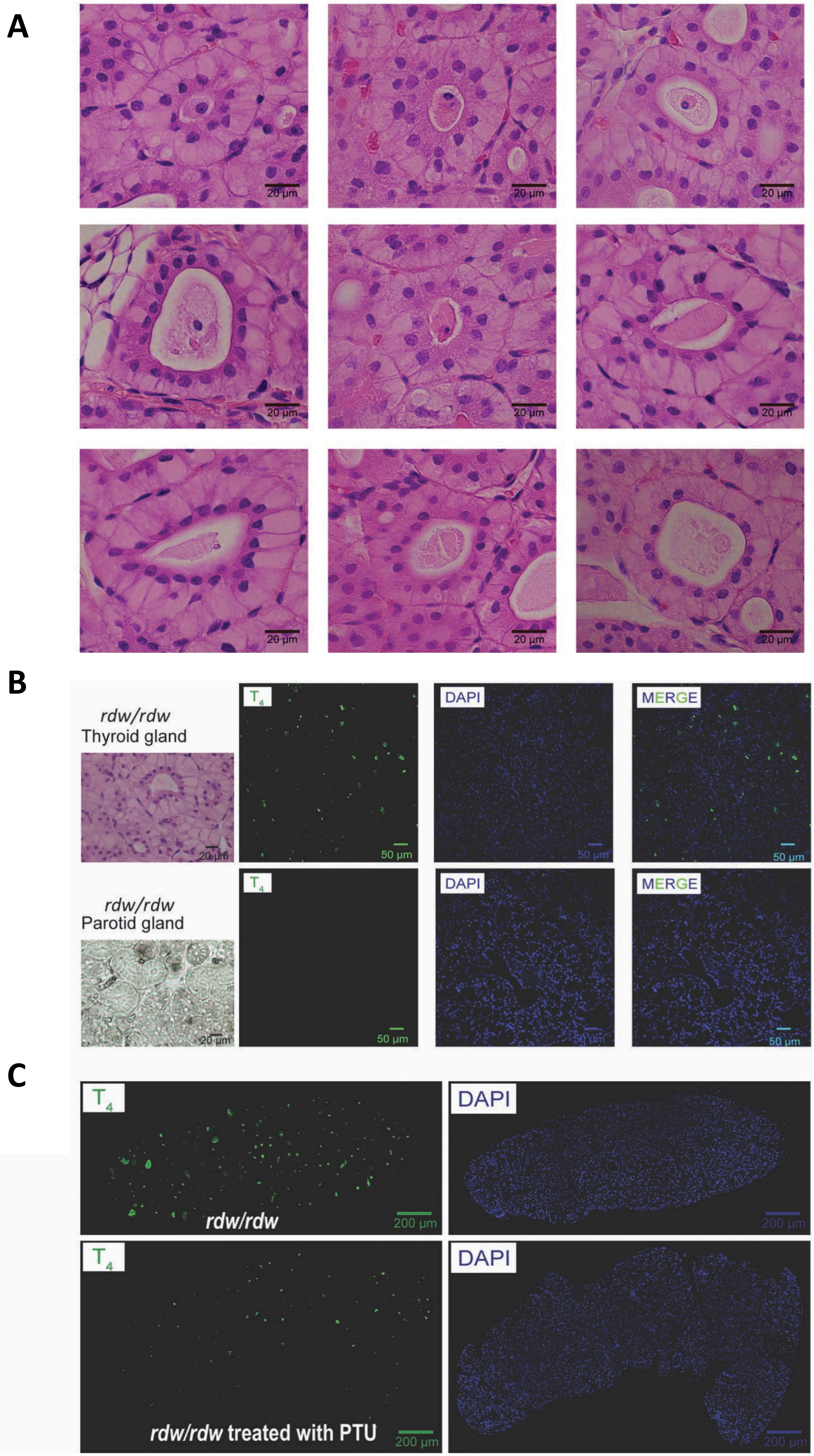

Zhang et al., Supplemental Fig. S3

A

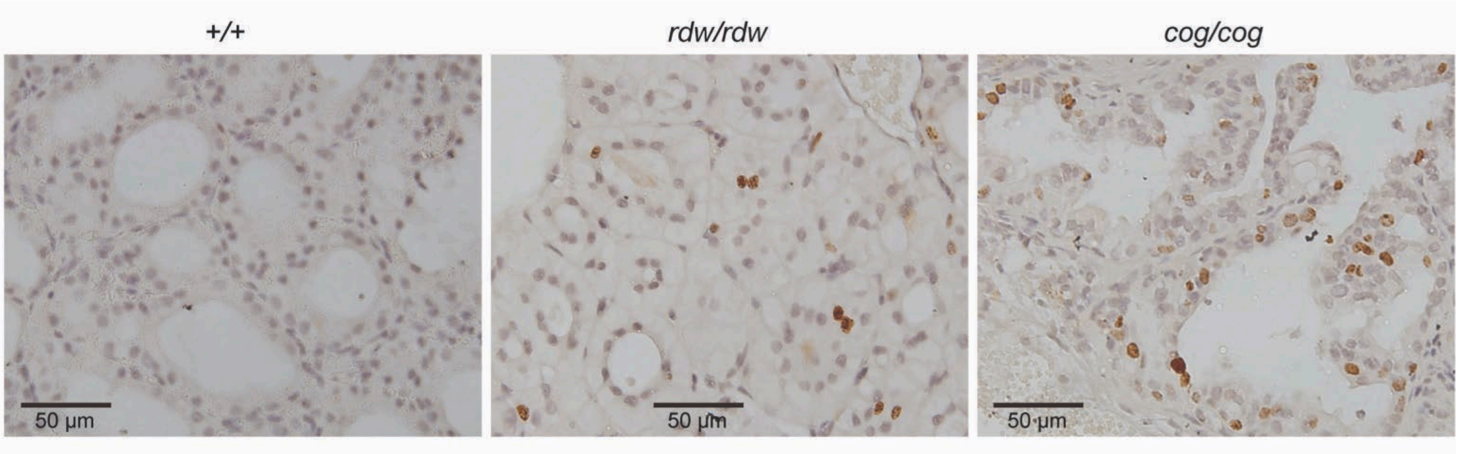

B

Normal Thyroid
Iodinated Tg in the follicle lumen

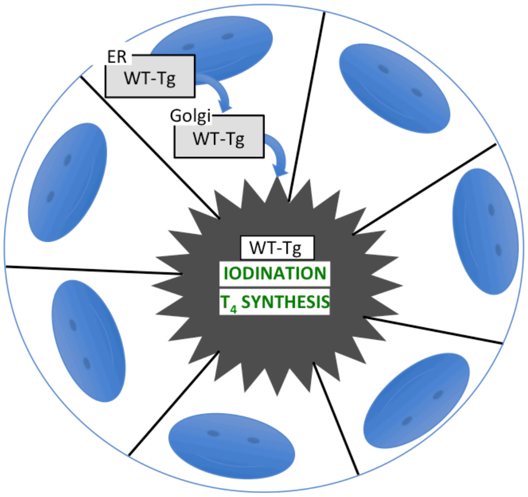

Homozygous mutant *TG* (e.g., *rdw/rdw* rat)
Tg entrapped in engorged ER; protein delivered to follicle lumen via cell death

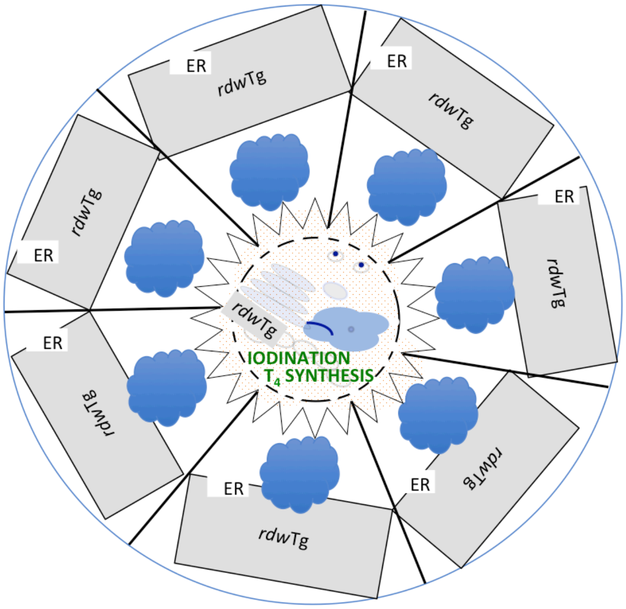

Zhang et al., Supplemental Fig. S4
